# Double Electroporation Combined with Zona Pellucida Removal Improves Biallelic Genome Editing Efficiency in Porcine Embryos

**DOI:** 10.3390/ani16121919

**Published:** 2026-06-20

**Authors:** Nanaka Torigoe, Takeshige Otoi, Manita Wittayarat, Oky Setyo Widodo, Theerawat Tharasanit, Kaywalee Chatdarong, Megumi Nagahara, Maki Hirata, Fuminori Tanihara, Zhao Namula

**Affiliations:** 1 College of Coastal Agricultural Sciences, Guangdong Ocean University, Zhanjiang 524088, China; torigoe.nanaka@otsuka.jp (N.T.); otoi@tokushima-u.ac.jp (T.O.); nagahara@tokushima-u.ac.jp (M.N.); mhirata@tokushima-u.ac.jp (M.H.); tanihara@tokushima-u.ac.jp (F.T.); 2 Bio-Innovation Research Center, Tokushima University, Tokushima 7793233, Japan; 3 Faculty of Veterinary Medicine, Universitas Airlangga, Surabaya 60115, Indonesia; oky.widodo@fkh.unair.ac.id; 4 Faculty of Veterinary Science, Chulalongkorn University, Bangkok 10330, Thailand; theerawat.t@chula.ac.th (T.T.); kaywalee.c@chula.ac.th (K.C.); 5 Faculty of Veterinary Science, Prince of Songkla University, Songkhla 90110, Thailand; mwittayarat@gmail.com

**Keywords:** double electroporation, gene editing, mosaic mutation, pig, zona free

## Abstract

The CRISPR/Cas9 system has been widely used for gene editing in various species; however, mosaicism remains a significant challenge. This study aimed to improve gene editing efficiency and reduce mosaicism in porcine embryos by exploring double electroporation pre- and post-in vitro fertilization combined with zona pellucida (ZP) removal. We evaluated the effects of these treatments on the development and mutation rates of oocytes/zygotes edited with guide RNAs (gRNAs) targeting *GGTA1*, *CMAH*, or *B4GALNT2* genes. Double electroporation significantly increased the total and biallelic mutation rates in ZP-intact zygotes but not in ZP-free zygotes edited using *GGTA1*-targeted gRNAs. All blastocysts from ZP-free zygotes exhibited biallelic mutations following double electroporation. For the *CMAH* gene, all blastocysts exhibited mutations (biallelic mutations ≥ 80%); however, double electroporation and ZP removal did not affect their mutation rates or efficiency. For the *B4GALNT2* gene, double electroporation significantly increased total mutation rates in ZP-intact zygotes, whereas all blastocysts from ZP-free zygotes showed biallelic mutation. These findings suggest that double electroporation, particularly with ZP removal, may enhance gene-editing efficiency, reduce mosaicism and improve the success of genetic modifications.

## Simple Summary

The CRISPR/Cas9 system has been successfully used for gene editing, but mosaicism is a challenge. This study aimed to enhance gene-editing efficiency and reduce mosaicism in porcine embryos using double electroporation before and after in vitro fertilization with zona pellucida (ZP) removal. We assessed treatments on oocytes/zygotes edited with gRNAs targeting the *GGTA1*, *CMAH*, or *B4GALNT2* genes. Double electroporation increased the total and biallelic mutation rates in ZP-intact zygotes edited with *GGTA1*. All blastocysts from ZP-free zygotes had biallelic mutations after double electroporation. For *CMAH*, all blastocysts had mutations, with over 80% biallelic, but double electroporation and ZP removal did not affect the rates. For *B4GALNT2*, double electroporation increased the mutation rates in ZP-intact zygotes, whereas all blastocysts from ZP-free zygotes showed biallelic mutations. These findings suggest that double electroporation, particularly with ZP removal, may enhance gene-editing efficiency, reduce mosaicism, and improve the efficiency of genetic modifications. This approach advances reproductive biotechnology by facilitating the generation of genetically modified pigs for biomedical applications.

## 1. Introduction

Pigs serve as valuable research models for studying human disease mechanisms and advancing gene therapy. Their anatomical and physiological resemblance to humans makes them a key source of cells, tissues, and organs for xenotransplantation [1]. The clustered regularly interspaced short palindromic repeats (CRISPR)/CRISPR-associated protein (Cas) system has been successfully utilized for gene editing in various mammalian species [2]. However, the high incidence of mosaicism in gene-edited founders remains a major obstacle to the efficient application of CRISPR/Cas gene editing technology [3]. Mosaicism occurs when an individual has multiple cell lineages with distinct genotypes, which can arise naturally during early embryonic development owing to DNA replication errors [4]. It can arise when CRISPR/Cas9-mediated DNA cleavage and subsequent repair occur after the first embryonic DNA replication or persist during successive cleavage divisions, resulting in genetically distinct cell lineages within a single embryo [5]. Thus, the earlier delivery of CRISPR/Cas9 components has been proposed to increase the likelihood of genome editing before DNA replication, which may contribute to reducing mosaicism [5,6]. Typically, the CRISPR system is introduced after fertilization to allow efficient editing before DNA replication [5,7,8]. However, delayed editing increases the likelihood of mosaicism at CRISPR-Cas9 target sites [9], potentially causing uneven editing across embryonic cells, resulting in genetically mosaic offspring [3]. Conversely, the CRISPR/Cas9 system has the potential to induce mutations during meiotic maturation in porcine oocytes, aligning with chromosomal condensation in preparation for fertilization [10,11]. Applying the CRISPR/Cas9 system during oocyte maturation may provide better control over gene mutations than applying it at later developmental stages. This approach allows gene editing during fertilization, leading to continued modifications of the paternal genome and multiple mutated alleles from both maternal and paternal copies [12,13]. Therefore, to enhance the impact of gene editing in biomedical research, approaches that minimize mosaicism must be developed and effectively integrated into advanced embryo manipulation and transfer systems.

Electroporation, a simple and efficient gene transfection method, is widely used to deliver the CRISPR system into various cell types with differing success rates [14]. We previously demonstrated the effectiveness of electroporation in inducing single genome mutations after introducing Cas9 protein into porcine embryos [15,16,17]. We also showed that the efficiency of single gene editing in blastocysts derived from oocytes electroporated with the CRISPR/Cas9 system before in vitro fertilization (IVF) was comparable to that in zygotes electroporated 13 h after IVF initiation [10]. Thus, achieving biallelic mutagenesis requires providing sufficient time for the CRISPR/Cas9 system to act either before or immediately after the first cleavage divisions [10]. However, our subsequent study revealed that electroporation prior to fertilization did not increase the overall biallelic mutation rates in the resulting blastocysts [18], highlighting the need for alternative strategies to improve gene-editing efficiency. Additionally, the zona pellucida (ZP), a glycoprotein layer surrounding the oocyte’s plasma membrane, acts as a protective barrier [19] and impedes the efficient delivery of CRISPR/Cas9 components during electroporation despite its vital role in fertilization and early embryonic development [20]. Thus, the ZP further limits the effectiveness of gene editing [21].

Although electroporation before fertilization and zona pellucida manipulation have been investigated separately in porcine embryos [18,21], no study has evaluated double electroporation spanning fertilization together with ZP removal to enhance genome-editing efficiency and reduce mosaicism. We hypothesized that repeated delivery of CRISPR/Cas9 ribonucleoprotein complexes before and after fertilization, combined with improved embryo access through ZP removal, would increase the efficiency of biallelic genome editing. Therefore, in this study, we aimed to improve gene editing efficiency and enhance biallelic mutation rates by exploring double electroporation, both before and after IVF, along with ZP removal from oocytes/zygotes. We examined the effects of these treatments on the development and mutation rates of oocytes/zygotes edited with guide RNAs (gRNAs) targeting the *GGTA1*, *CMAH*, and *B4GALNT2* genes. These genes were selected as targets because they encode major carbohydrate xenoantigens implicated in hyperacute and antibody-mediated rejection in pig-to-human xenotransplantation. Efficient disruption of these genes is a key strategy for reducing xenogeneic antigenicity and improving xenograft compatibility. This study provides an appropriate model for evaluating the efficacy of novel genome-editing approaches for generating donor pigs for xenotransplantation. Moreover, this approach could refine gene editing techniques, reduce mosaicism, and enhance genetic modifications, advancing reproductive biotechnology and assisted embryo manipulation techniques in pigs.

## 2. Materials and Methods

### 2.1. Ethical Approval

All animal experiments were approved by the Institutional Animal Care and Use Committee of Tokushima University (approval number: T2019-11).

### 2.2. Oocyte Collection, In Vitro Maturation (IVM), Fertilization, and Embryo Culture

Pig ovaries were obtained from slaughtered prepubertal gilts (Landrace × Large White × Duroc breeds) at a local slaughterhouse. Cumulus-oocyte complexes (COCs) were collected under a stereomicroscope. Approximately 50 COCs were cultured in 500 µL of maturation medium for 22 h in four-well dishes (Nunc A/S, Roskilde, Denmark). The maturation medium consisted of tissue culture medium 199 with Earle’s salts (TCM 199; Invitrogen Co., Carlsbad, CA, USA) supplemented with 10% (*v*/*v*) porcine follicular fluid, 0.6 mM cysteine (Sigma-Aldrich, St. Louis, MO, USA), 50 µM sodium pyruvate (Sigma-Aldrich), 2 mg/mL D-sorbitol (FUJIFILM Wako Pure Chemical Industries Ltd., Osaka, Japan), 50 µM β-mercaptoethanol (FUJIFILM Wako Pure Chemical Industries Ltd.), 1 µg/mL 17 β-estradiol (Sigma-Aldrich), 10 IU/mL equine chorionic gonadotropin (Kyoritu Seiyaku, Tokyo, Japan), 10 IU/mL human chorionic gonadotropin (Kyoritu Seiyaku), and 50 µg/mL gentamicin (Sigma-Aldrich). The COCs were subsequently transferred into fresh IVM medium without hormones and cultured for 22 h at 39 °C in a humidified incubator containing 5% CO_2_ in air.

Matured oocytes were subjected to IVF as described previously [22]. Briefly, frozen–thawed ejaculated spermatozoa were transferred to 5 mL of fertilization medium (PFM; Research Institute for the Functional Peptides Co., Yamagata, Japan) and washed via centrifugation at 500× *g* for 5 min. The pelleted spermatozoa were resuspended in fertilization medium and adjusted to a concentration of 2 × 10^6^ cells/mL. Subsequently, approximately 50 oocytes were transferred to 500 µL of sperm-containing fertilization medium covered with mineral oil in four-well dishes and incubated for 5 h at 39 °C in a humidified incubator containing 5% CO_2_, 5% O_2_, and 90% N_2_. The final sperm concentration was adjusted to 1 × 10^6^ sperms/mL.

After IVF, the putative zygotes were transferred to porcine zygote medium (PZM-5; Research Institute for the Functional Peptides Co.) and cultured continuously in vitro for 3 days at 39 °C in a humidified incubator containing 5% CO_2_, 5% O_2_, and 90% N_2_ in 25-well plates (one embryo per 15 µL of culture medium; ART Culture Dish, Nipro, Osaka, Japan) [23]. The cleaved embryos were transferred to porcine blastocyst medium (PBM; Research Institute for Functional Peptides Co.) in 25-well plates and cultured for 4 days to assess their development into blastocysts and determine their genotype. No morphological evaluation of the blastocysts was performed; instead, we randomly selected blastocysts that had reached the blastocyst stage for subsequent genotyping.

### 2.3. Electroporation

gRNAs targeting *GGTA1*, *CMAH*, or *B4GALNT2*, which are key targets to reduce immune rejection in xenotransplantation, were prepared as previously described [24] (Table 1). Electroporation was performed as described previously [25]. Briefly, an electrode (LF501PT1-20; BEX, Tokyo, Japan) was connected to a CUY21EDIT II electroporator (BEX) and placed under a stereoscopic microscope. The oocytes and zygotes (approximately 40–50 in number) were aligned linearly in the electrode gap of a chamber slide filled with 10 μL of nuclease-free duplex buffer [Integrated DNA Technologies (IDT), Coralville, IA, USA]. The buffer contained 100 ng/μL of each gRNA (Alt-R CRISPR crRNAs and tracrRNA, chemically modified and length-optimized variants of the native gRNAs purchased from IDT) and 200 ng/μL Cas9 protein (Guide-it Recombinant Cas9; Takara Bio, Shiga, Japan). The samples were then electroporated using five 1 ms pulses at 25 V.

### 2.4. Analysis of Target Genes After Electroporation

We estimated the frequencies of base insertions and deletions (indels) in the target regions of individual blastocysts to analyze the efficiency of introducing target mutations in the embryos. The genomic DNA of individual blastocysts was extracted by heat treatment with 50 mM NaOH. After neutralization, the DNA samples were subjected to polymerase chain reaction (PCR) using KOD One PCR Master Mix (Toyobo, Osaka, Japan) according to the manufacturer’s instructions. The primers used for the amplification are listed in Table 1. After purification of PCR products using Fast Gene Gel/PCR Extraction Kit (Nippon Genetics, Tokyo, Japan), the sequences of the target regions were analyzed by Sanger sequencing using a BigDye Terminator Cycle Sequencing Kit version 3.1 (Thermo Fisher Scientific, Inc., Tokyo, Japan) on an ABI 3500 genetic analyzer (Applied Biosystems, Foster City, CA, USA). Sequencing was performed on several blastocysts; however, only those with successful sequencing results were included in the subsequent analysis. The Tracking of Indels by DEcomposition (TIDE; https://tide.nki.nl) bioinformatics package was used to analyze the Sanger sequencing data (Supplementary Figure S1). Sequence quality was assessed based on chromatogram signal clarity, and only sequences with consistently clear peak resolution across the entire trace were used for the TIDE analysis. Sequence traces were aligned to a wild-type reference sequence using the default alignment parameters. The decomposition window was set to 10–50 bp downstream of the expected Cas9 cleavage site. Indels within a size range of up to ±30 bp were analyzed. The quality of the decomposition was assessed using the coefficient of determination (*R*^2^) and only results with *R*^2^ ≥ 0.9 were considered for further analysis. Blastocysts were classified as biallelic mutants when no wild-type sequence signal was detected (wild-type frequency < 5%). Blastocysts were classified as mosaics when multiple indel sequences were detected in combination with residual wild-type sequences (wild-type frequency ≥ 5%). Blastocysts were classified as wild-type when only the wild-type sequence was detected without indel signals. The editing rate was defined as the ratio of the number of gene-edited blastocysts to the total number of sequenced blastocysts. Editing efficiency was defined as the proportion of indel mutation events in blastocysts carrying the mosaic or biallelic mutation products. Off-target analyses were not performed in this study.

### 2.5. Experimental Design

**Figure 1:**
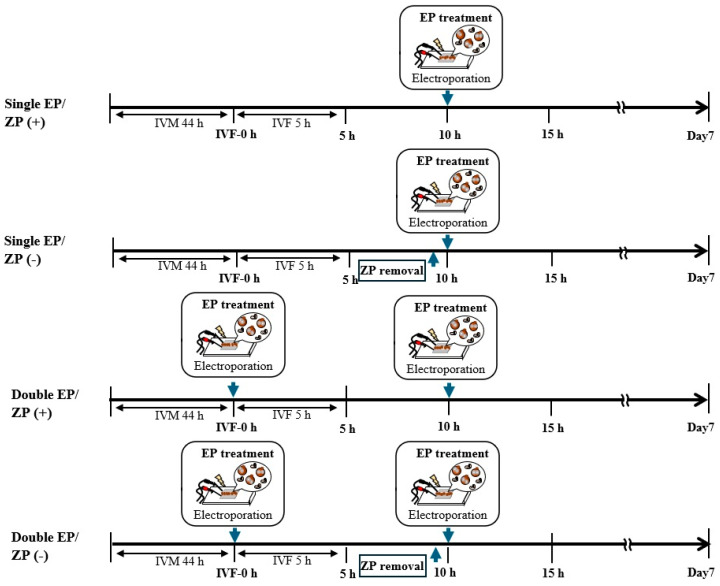
Schematic representation of the experimental design. Porcine oocytes were subjected to in vitro maturation (IVM) for 44 h and assigned to four treatment groups: single electroporation (EP) with zona pellucida (ZP) intact (+), single EP with ZP removal (−), double EP with ZP intact (+), and double EP with ZP removal (−). In the single-EP groups, electroporation of Cas9-gRNA ribonucleoprotein (RNP) complexes was performed 10 h after in vitro fertilization (IVF). In the double-EP groups, electroporation was performed immediately before IVF and again 10 h after IVF. Embryos were cultured to Day 7, and blastocyst development and genome-editing outcomes were evaluated. The experimental procedure was performed using gRNAs targeting three xenotransplantation-related genes: *GGTA1*, *CMAH*, and *B4GALNT2*. IVM, in vitro maturation; IVF, in vitro fertilization; EP, electroporation; ZP, zona pellucida.

To examine the effects of double electroporation and ZP removal on the development and mutation of oocytes/zygotes edited with gRNAs targeting *GGTA1*, *CMAH*, or *B4GALNT2*, electroporation was performed on oocytes/zygotes with and without ZP, either once (single) or twice (double), using each gRNA separately (Figure 1). For single electroporation, the process was performed 10 h after the initiation of IVF on zygotes with and without ZP. For double electroporation, ZP-intact oocytes were first electroporated immediately before IVF and then again 10 h after IVF initiation on zygotes with and without ZP. To assess the effect of ZP removal, a subset of zygotes was treated with 0.5% (*w*/*v*) actinase-E (Kaken-Seiyaku Corp., Tokyo, Japan) in Dulbecco’s phosphate-buffered saline (Nissui Pharmaceutical, Tokyo, Japan) for 20–30 s, transferred to PZM-5 without actinase-E, and gently pipetted to completely remove their ZP. After actinase-E treatment, the zygotes were examined under a microscope to confirm complete ZP removal. These ZP-free zygotes were then subjected to electroporation according to their assigned protocol (single or double electroporation). All zygotes were subsequently cultured in 25-well plates (one embryo per 15 µL of culture medium) for 7 days [23]. Five independent replicates were performed for each gRNA.

### 2.6. Statistical Analysis

The percentage data for embryo development and mutation efficiency were analyzed using analysis of variance (ANOVA) with the general linear model procedure in SAS for Windows, version 9.1 (SAS Institute, Cary, NC, USA). Percentage data were subjected to arcsine square-root transformation before statistical analysis. The normality of the data distribution was evaluated using the Jarque–Bera test before performing ANOVA. The statistical model included ZP removal, electroporation treatment, and two-way interactions. Non-significant interactions were excluded from the final model; however, the main effects of ZP removal and electroporation treatment were still tested in the refined model. The percentages of gene-edited blastocysts were analyzed using the chi-square test. Differences with a *p*-value (*p*) of 0.05 or less were considered statistically significant.

## 3. Results

The double electroporation or ZP removal treatments did not affect the blastocyst formation rates of zygotes edited using gRNAs targeting *GGTA1*, *CMAH*, or *B4GALNT2*. Upon editing with the gRNA targeting *GGTA1* (Table 2), the double electroporation treatments significantly increased the total and biallelic mutation rates of ZP-intact zygotes (*p* < 0.046) but not of ZP-free zygotes compared with the single electroporation treatment. However, all blastocysts from ZP-free zygotes showed biallelic mutation after double electroporation. ZP removal significantly increased the mutation efficiency of gene-edited blastocysts (*p* < 0.009) in the single electroporation treatment group but not in the double electroporation treatment group.

Upon editing with the gRNA targeting *CMAH* (Table 3), all blastocysts edited were mutated, and >80% of the embryos had biallelic mutations. However, double electroporation or ZP removal had no effect on the mutation rates and mutation efficiency of these blastocysts.

Upon editing with the gRNA targeting *B4GALNT2* (Table 4), double electroporation significantly increased the total mutation rates of ZP-intact zygotes (*p* < 0.034) but not of ZP-free zygotes. Moreover, all blastocysts from ZP-free zygotes subjected to double electroporation exhibited biallelic mutation.

## 4. Discussion

In this study, we introduced the CRISPR/Cas9 system into presumptive zygotes 10 h after IVF in the single electroporation group. This timing corresponds to the period between fertilization and the start of genome replication, a window lasting about 12 to 14 h in pigs [7], which is considered optimal for CRISPR/Cas9 delivery into embryos [8]. Our results support this approach, as no significant effect of double electroporation or ZP removal was observed in zygotes electroporated with gRNA targeting the *CMAH* gene, which had already shown high genome editing efficiency (biallelic mutations > 80%) with single electroporation. As the genome editing efficiency with single electroporation was already high, the additional introduction of CRISPR/Cas9 via double electroporation might not yield improved results. However, the single-electroporation approach was less effective for the other two genes, *GGTA1* and *B4GALNT2*, suggesting that the choice of target gene influences editing success. In these cases, double electroporation improved gene editing efficiency. This gene-dependent variation may be attributed to factors such as gRNA sequence efficiency, chromatin accessibility, and locus-specific repair dynamics [26,27,28]. Recent studies of porcine embryos also showed variation in genome-editing outcomes across target genes and delivery strategies, indicating locus-specific and procedural effects on efficiency [6,29]. However, the mechanisms responsible for the gene-dependent differences in editing efficiency remain unclear. Potential contributing factors include gRNA sequence characteristics, such as secondary structure and GC content, as well as locus accessibility at the target sites, but these parameters were not evaluated in the present study.

Although CRISPR/Cas9 is typically introduced after fertilization and before DNA replication for efficient editing, applying it before fertilization offers an effective alternative. Our results showed that double electroporation significantly improved the biallelic mutation rates in ZP-intact zygotes electroporated with the gRNA targeting *GGTA1*, whereas it improved the total mutation rate in those electroporated with the *B4GALNT2*. The improvement in biallelic mutation rates may be associated with the combined effects of electroporation timing and repeated delivery of CRISPR/Cas9 components via the double-electroporation procedure. The first electroporation was performed before IVF initiation, and the second was performed 10 h after IVF, which may have increased the opportunity for CRISPR/Cas9 ribonucleoprotein complexes to enter the embryos before the first DNA replication. However, our previous study demonstrated that electroporation of mature oocytes at 44 h of IVM resulted in mutation and biallelic mutation rates comparable to those obtained after electroporation of putative zygotes 13 h after IVF [18]. Similarly, we recently reported that electroporation performed before IVF and at 5 and 10 h post-IVF produced comparable rates of mutation and biallelic mutations in porcine embryos [29]. These findings suggest that electroporation timing alone is unlikely to fully explain the improved editing outcomes observed in this study. Piñeiro-Silva and Gadea [30] summarized evidence indicating that the developmental stage at which CRISPR/Cas9 components are introduced may influence genome-editing outcomes, although the optimal timing remains unknown. Navarro-Serna et al. [6] demonstrated that electroporation of in vitro-matured oocytes before IVF efficiently generated single-, double-, and quintuple-gene knockout embryos without compromising their blastocyst development. In particular, simultaneous mutations were successfully introduced into multiple xenotransplantation-related genes, including *GGTA1*, *B4GALNT2*, and *CMAH* genes. Taken together, these findings suggest that electroporation before fertilization may provide additional opportunities for CRISPR/Cas9 activity during early embryogenesis. However, the optimal timing and its independent contribution to editing efficiency remain unclear. Therefore, the improved editing outcomes observed in the present study are more likely attributable to the combined effects of repeated electroporation and electroporation timing than to timing alone. The double electroporation strategy, spanning the pre- and post-fertilization periods, may increase opportunities for CRISPR/Cas9 activity during zygotic genome establishment and the onset of gene expression in the embryo. Although multiplex genome editing was not investigated in the present study, the improved biallelic mutation rates observed here suggest that double electroporation combined with zona pellucida removal warrants further evaluation.

In mice, pre-treatment with Tyrode’s acidic solution has been used to increase ZP permeability, allowing larger RNA molecules to enter during electroporation [31,32]. In our previous study in pigs, weakening the ZP through actinase E treatment did not alter the developmental competence, mutation rate, or mutation efficiency of electroporated zygotes [21]. However, when using double electroporation, its potential adverse effects on embryo development must be considered. Although detrimental impacts, including reduced blastocyst rates, have been reported previously [33], we found no such effect on blastocyst formation even after ZP removal, consistent with the findings from another study [34]. However, for practical applications, particularly in large-scale embryo transfers, improvements to the procedure and optimization of the culture system may be necessary. On the other hand, in zygotes electroporated with the gRNA targeting *B4GALNT2*, the biallelic mutation rate was significantly enhanced to reach 100% by combining double electroporation and ZP removal. Thus, this combination of treatments, particularly for genes with low editing efficiency, may enhance CRISPR/Cas9 delivery into fertilized zygotes and may reduce mosaicism. By performing the first electroporation before IVF initiation and the second electroporation 10 h after IVF initiation, ribonucleoproteins may have been delivered over a broader developmental window before the first DNA replication. Additionally, ZP removal may have improved the delivery of the CRISPR/Cas9 ribonucleoprotein complex into zygotes, further improving gene editing efficiency [21]. Our findings suggest that optimizing the timing of CRISPR/Cas9 delivery may be important for efficient genome editing. Overcoming physical barriers to ribonucleoprotein entry may also improve editing outcomes.

A limitation of the present study is that the fertilization rate and polyspermy incidence were not evaluated. Because electroporation before IVF may influence sperm penetration and fertilization dynamics, these effects remain unclear. Additionally, although significant differences were detected in several comparisons, the relatively small sample size in some groups may have reduced the statistical power to detect more subtle effects. The number of blastocysts available for mutation analysis was further reduced by sequencing failures, which occurred at similar frequencies across the experimental groups. Another limitation is that the mutation analysis used TIDE based on Sanger sequencing data. Although TIDE is a widely used approach for estimating mutation frequencies and identifying indel patterns, it may underestimate the complexity of mosaic mutations and low-frequency alleles. In addition, the definitions of biallelic mutations and mosaicism in the present study were based on TIDE-derived estimates of wild-type and mutant sequence frequencies. Therefore, complex editing patterns, including compound heterozygous mutations and low-frequency mosaic alleles, may not have been completely resolved. Accordingly, the mutation profiles and classifications reported here should be interpreted within the limitations of this analytical method. Although no adverse effects on blastocyst development were observed, subtle cellular stress responses or epigenetic alterations induced by repeated electroporation and zona pellucida removal cannot be excluded. Moreover, embryo quality was assessed only by blastocyst formation rates; indicators such as total cell number and apoptosis were not evaluated. Thus, the effects of repeated electroporation may have gone undetected. Because blastocyst formation alone may not fully reflect the biological consequences of embryo manipulation, the potential effects on cellular physiology, gene expression, and epigenetic status remain unknown. Furthermore, although a previous report demonstrated that CRISPR/Cas9 delivery results in lower off-target mutation rates owing to rapid protein turnover [35], off-target effects were not evaluated in the present study, and no in silico prediction of off-target sites was performed. In addition, the present study evaluated genome-editing outcomes only up to the blastocyst stage. Another limitation is that mosaicism was inferred from whole-blastocyst sequencing profiles rather than evaluated at the single-cell level. Therefore, further studies are required to comprehensively evaluate these approaches. Such studies should include targeted amplicon or deep sequencing, in silico and experimental off-target analyses, single-cell genotyping, embryo transfer, and offspring production.

## 5. Conclusions

The combination of double electroporation and ZP removal significantly enhances CRISPR/Cas9 editing efficiency, particularly for *B4GALNT2*, which is typically challenging to edit. Optimized electroporation timing may facilitate the delivery of CRISPR/Cas9 components before DNA replication, thereby reducing mosaicism. ZP removal further improves gene-editing efficiency without compromising embryo development, making this approach a promising method for enhancing genome editing in porcine models. By combining double electroporation across the fertilization stage with ZP removal, this study may provide a strategy to reduce mosaicism while maintaining embryo development.

## Figures and Tables

**Table 1:** gRNA and primer sequences used for sequencing analysis *.

Target Gene	gRNA			Primer	
	Target Sequence	PAM	Strand	Forward Primer	Reverse Primer
*GGTA1*	AGACGCTATAGGCAACGAAA	AGG	Sense	AAAAGGGGAGCACTGAACCT	CCTGTCGGGAATGTTCTCAT
*CMAH*	GAAGCTGCCAATCTCAAGGA	AGG	Sense	GCTGTCAATGCTCAGGGATT	TGCCAAACCTAATTGGGAGA
*B4GALNT2*	TTGAGGATCGACAGACATCT	AGG	Antisense	GACCAGACATCGTTCCCAGT	GGGAACTGGCTGTAAAGTGG

* Based on NCBI: Sus scrofa isolate TJ Tabasco breed Duroc, whole genome shotgun sequence, Sscrofa11. 1 (GCF_000003025.5). *GGTA1* = Alpha-1,3-galactosyltransferase, *CMAH* = Cytidine-monophosphate-N-acetylneuraminic acid hydroxylase, *B4GALNT2* = β-1,4-N-acetyl-galactosaminyltransferase 2.

**Table 2:** Effects of double electroporation and zona pellucida (ZP) removal on the development and mutation rates of oocytes/zygotes edited using the gRNA targeting *GGTA1* *.

Electroporation Treatment **	Zona Pellucida	No. of Oocytes Examined	No. (%) of Embryos Developed to Blastocysts	No. of Blastocysts Examined	No. (%) of Gene-Edited Embryos ***			Mutation Efficiency ****
					Total Mutation	Mosaic	Biallelic	
Single	+	230	36 (15.8 ± 2.7)	19	14 (73.7) ^a^	6 (31.6) ^a^	8 (42.1) ^a^	77.5 ± 6.6 ^a^
Single	−	233	42 (18.2 ± 3.4)	17	17 (100) ^b^	1 (5.9) ^a,b^	16 (94.1) ^b^	91.2 ± 1.2 ^b^
Double	+	224	37 (16.6 ± 3.8)	19	19 (100) ^b^	2 (10.5) ^a,b^	17 (89.5) ^b^	86.5 ± 4.4 ^a,b^
Double	−	233	35 (15.1 ± 3.8)	19	19 (100) ^b^	0 (0) ^b^	19 (100) ^b^	92.0 ± 0.2 ^b^

* Five replicate trials were performed. Percentages are expressed as mean ± standard error of the mean (SEM). ** Single electroporation was applied to zygotes with (+) and without (−) ZP after 10 h of in vitro fertilization (IVF) initiation. Double electroporation was applied to ZP-intact oocytes immediately before IVF and then to zygotes with (+) and without (−) ZP after 10 h of IVF. *** Percentages were calculated by dividing the number of gene-edited blastocysts by the number of examined blastocysts. **** The mutation efficiency represents the proportion (mean ± SEM) of indel mutation events in mutant blastocysts determined by the TIDE analysis. ^a,b^ Values with different superscript letters in the same column are significantly different (*p* < 0.05).

**Table 3:** Effects of double electroporation and zona pellucida (ZP) removal on the development and mutation rates of oocytes/zygotes edited using the gRNA targeting *CMAH* *.

Electroporation Treatment **	Zona Pellucida	No. of Oocytes Examined	No. (%) of Embryos Developed to Blastocysts	No. of Blastocysts Examined	No. (%) of Gene-Edited Embryos ***			Mutation Efficiency ****
					Total Mutation	Mosaic	Biallelic	
Single	+	227	31 (13.5 ± 1.6)	16	16 (100)	3 (18.8)	13 (81.3)	85.9 ± 6.4
Single	−	216	34 (15.7 ± 2.5)	16	16 (100)	2 (12.5)	14 (87.5)	93.3 ± 3.0
Double	+	222	35 (15.8 ± 2.4)	20	20 (100)	1 (5.0)	19 (95.0)	95.9 ± 0.9
Double	−	212	32 (15.2 ± 2.0)	18	18 (100)	1 (5.6)	17 (94.4)	95.6 ± 1.6

* Five replicate trials were performed. Percentages are expressed as mean ± standard error of the mean (SEM). ** Single electroporation was applied to zygotes with (+) and without (−) ZP after 10 h of in vitro fertilization (IVF) initiation. Double electroporation was applied to ZP-intact oocytes immediately before IVF and then to zygotes with (+) and without (−) ZP after 10 h of IVF. *** Percentages were calculated by dividing the number of gene-edited blastocysts by the number of examined blastocysts. **** The mutation efficiency represents the proportion (mean ± SEM) of indel mutation events in mutant blastocysts determined by the TIDE analysis.

**Table 4:** Effects of double electroporation and zona pellucida (ZP) removal on the development and mutation rates of oocytes/zygotes edited using the gRNA targeting *B4GALNT2* *.

Electroporation Treatment **	Zona Pellucida	No. of Oocytes Examined	No. (%) of Embryos Developed to Blastocysts	No. of Blastocysts Examined	No. (%) of Gene-Edited Embryos ***			Mutation Efficiency ****
					Total Mutation	Mosaic	Biallelic	
Single	+	233	28 (11.5 ± 1.9)	13	9 (69.2) ^a^	2 (15.4)	7 (53.8) ^a^	85.5 ± 5.2
Single	−	224	22 (9.0 ± 2.2)	13	12 (92.3) ^a,b^	0 (0)	12 (92.3) ^a,b^	94.4 ± 0.9
Double	+	215	20 (10.1 ± 2.8)	15	15 (100) ^b^	2 (13.3)	13 (86.7) ^a,b^	84.9 ± 6.8
Double	−	198	12 (7.4 ± 2.6)	9	9 (100) ^a,b^	0 (0)	9 (100) ^b^	93.7 ± 1.0

* Five replicate trials were performed. Percentages are expressed as mean ± standard error of the mean (SEM). ** Single electroporation was applied to zygotes with (+) and without (−) ZP after 10 h of in-vitro fertilization (IVF) initiation. Double electroporation was applied to ZP-intact oocytes immediately before IVF and then to zygotes with (+) and without (−) ZP after 10 h of IVF. *** Percentages were calculated by dividing the number of gene-edited blastocysts by the number of examined blastocysts. **** The mutation efficiency represents the proportion (mean ± SEM) of indel mutation events in mutant blastocysts determined by the TIDE analysis. ^a,b^ Values with different superscript letters in the same column are significantly different (*p* < 0.05).

## Data Availability

The data used to support the findings of this study have been included in this article.

## Supplementary Materials

The following supporting information can be downloaded at: https://www.mdpi.com/article/10.3390/ani16121919/s1, Figure S1: Representative Sanger sequencing chromatograms and corresponding TIDE decomposition outputs used for the classification of wild-type, mosaic, and biallelic mutations in blastocysts edited with gRNAs targeting *GGTA1*, *CMAH*, and *B4GALNT2*.

## Abbreviations

The following abbreviations are used in this manuscript:

CRISPR	clustered regularly interspaced short palindromic repeats
Cas	CRISPR-associated protein
COCs	cumulus-oocyte complexes
IVF	in vitro fertilization
IVM	in vitro maturation
gRNA	guide RNA
PBM	porcine blastocyst medium
PCR	polymerase chain reaction
PZM	porcine zygote medium
SEM	standard error of the mean
ZP	zona pellucida
