# Supplementary figures and images for: Double Electroporation Combined with Zona Pellucida Removal Improves Biallelic Genome Editing Efficiency in Porcine Embryos
Source: Animals (Basel). 2026 Jun 20;16(12):1919. doi: 10.3390/ani16121919 (PMC13295827; doi:10.3390/ani16121919)

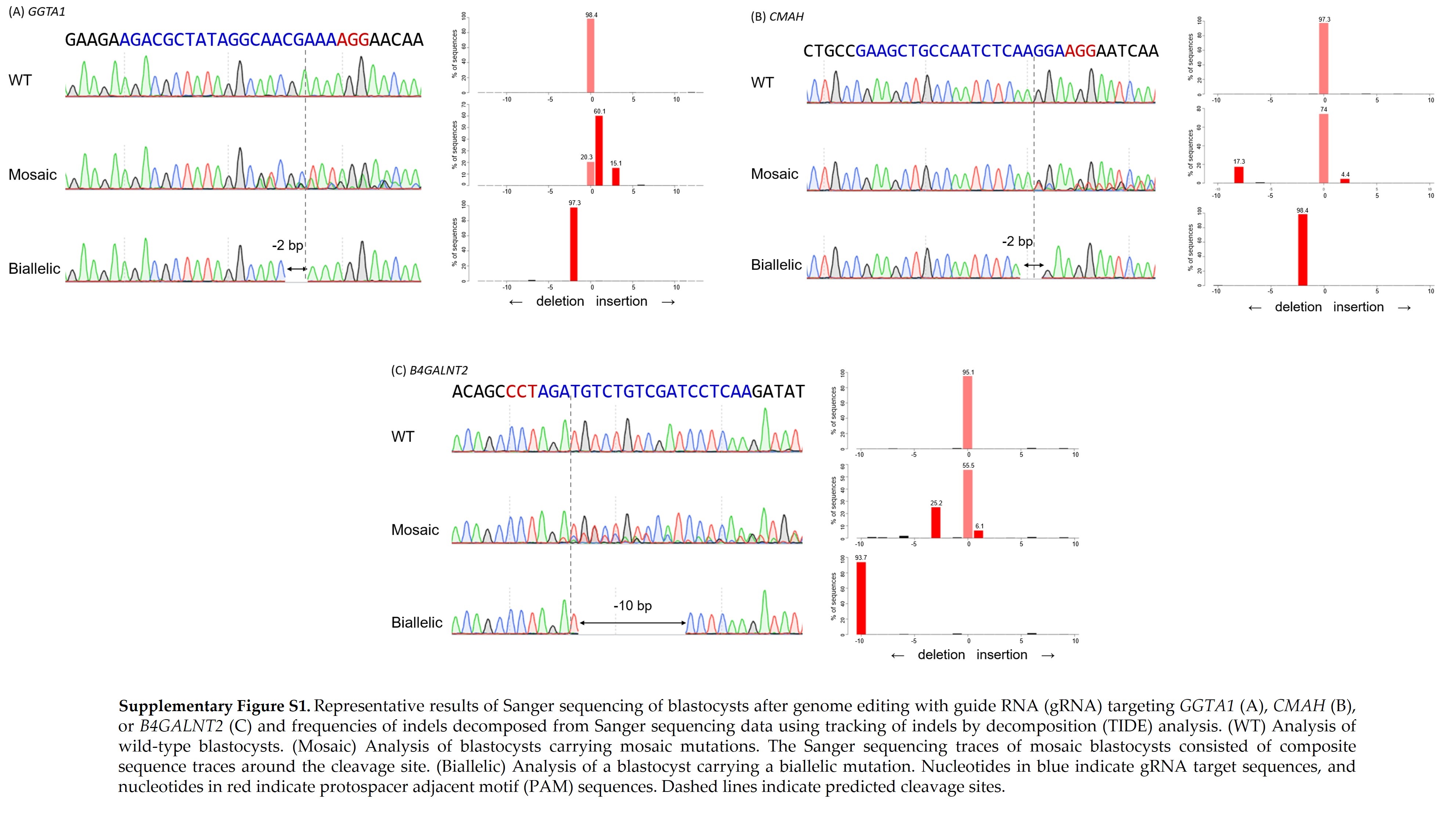

Supplement: Supplementary file 1 [file animals-16-01919-s001.zip › animals-4345114-supplementary.jpg]
